# P05.71. A social science review of the TCM/Oriental medicine literature: initial lessons

**DOI:** 10.1186/1472-6882-12-S1-P431

**Published:** 2012-06-12

**Authors:** T Stuardi, C Cassidy, L Conboy, V Kumar

**Affiliations:** 1 Social Science Review Project, Fairhope, USA; 2 New England School of Acupuncture, Newton, USA; 3 Johns Hopkins University, Baltimore, USA

## Purpose

The purpose of this project was to identify social science papers in the traditional Chinese medicine (TCM) and Oriental medicine (OM) literature. We believe the growing emphasis on qualitative research necessitates a review of the literature to date. By locating a compendium of articles that study Chinese medicine through social science, we aim to achieve two objectives. First, all of the material will be catalogued to provide a resource for fellow researchers. Second, papers on similar topics will be reviewed to produce qualitative meta-syntheses that can direct future research.

## Methods

We participated in several meetings to refine our definitions of social science, TCM, and OM, and to identify relevant search terms and databases. We used names of individual modalities related to TCM and OM (e.g. acupuncture), and broad methodological terms (e.g. qualitative research). Searches were conducted in MEDLINE, AMED, AnthroSource, PsycINFO, Dissertation Index and Social Sciences Citation Index, from 1990 – January 2012. Reference lists of relevant papers and book chapters were also hand-searched.

## Results

Difficulties in accessing social science research arose from the lack of indexing of particular journals (e.g. European Journal of Oriental Medicine), the absence of keywords, keyword variations, and shared keywords with biomedical conditions. For example, the search attempting to identify papers concerning Chinese medicine cupping found optic nerve cupping associated with glaucoma in biomedicine. Over five thousand papers were identified in the searches, and were subsequently assessed for relevance. Approximately two hundred papers will be catalogued and included in future meta-syntheses.

## Conclusion

Various difficulties are associated with identifying social science studies related to Chinese medicine; meanwhile, the number of studies increases. As Chinese medicine continues to grow as a source of integrative care, authors need to ensure that their work is accessible to the research community, by employing clear keywords and publishing in indexed journals.

